# The states of senescent cells

**DOI:** 10.1042/BST20253054

**Published:** 2025-08-04

**Authors:** Laura Boose de Mendonça, Guido Lenz, Eduardo Cremonese Filippi-Chiela

**Affiliations:** 1 Centro de Biotecnologia, Universidade Federal do Rio Grande do Sul, Porto Alegre, Rio Grande do Sul 91501-970, Brazil; 2 Programa de Pós-Graduação em Biologia Celular e Molecular, Universidade Federal do Rio Grande do Sul, Porto Alegre, Rio Grande do Sul 91501-970, Brazil; 3 Centro de Pesquisa Experimental, Hospital de Clínicas de Porto Alegre, Porto Alegre, Rio Grande do Sul 90035-903, Brazil; 4 Departamento de Biofísica, IB, Universidade Federal do Rio Grande do Sul, Porto Alegre, Rio Grande do Sul 91501-970, Brazil; 5 Departamento de Ciências Morfológicas, Universidade Federal do Rio Grande do Sul, Porto Alegre, Rio Grande do Sul 90050-170, Brazil

**Keywords:** cell state, cellular senescence, phenotypic dynamics, phenotypic heterogeneity, senescence-associated secretory phenotype

## Abstract

Senescent cells (SnCs) have typical changes in multiple features, such as increased cellular and nuclear size, morphofunctional alterations in organelles, and high secretory activity. The literature generally groups cellular changes and the non-proliferative character of SnCs into the autonomous senescent phenotype. In contrast, the influence of molecules and extracellular vesicles secreted by SnCs characterizes their non-autonomous phenotype. Unlike the detailed characterization of the structure of SnCs, the discussion regarding SnC states, which are characterized by the comprehensive integration of multiple features a cell harbors in a given moment, is still incipient. This review discusses the possible SnC states (SenStates) and their influence in pathophysiological contexts. We also discuss the main mechanisms and molecular players involved in the establishment and dynamics of these states, such as transcription factors, epigenetic marks, chromatin structure, and others. Finally, we discuss the biological relevance and potential clinical applications of SenStates, as well as open questions in the field.

## Cellular senescence

Cellular senescence is characterized by permanent arrest in cell division due to replicative stress driven by telomere erosion or prematurely due to damage to the genome, organelles, or cytoskeleton. Premature senescence can also be induced by oncogene overactivation (oncogene-induced senescence, OIS), loss of tumor suppressor genes, oxidative stress, epigenetic modulation, or Cyclin-Dependent Kinase Inhibitor (CDK inhibitors). Senescence-inducing stresses activate intracellular pathways like DNA damage response, Tumor Protein p53 (TP53), and Mitogen-Activated Protein Kinase (MAPK), ultimately leading to the increase in the expression of senescence effectors, such as CDKN1A (encoding the CDK inhibitor p21) and CDKN2A (encoding the CDK inhibitor p16INK4A and the Mdm2 inhibitor p14ARF), thus blocking the cell cycle [1].

**Figure 1:**
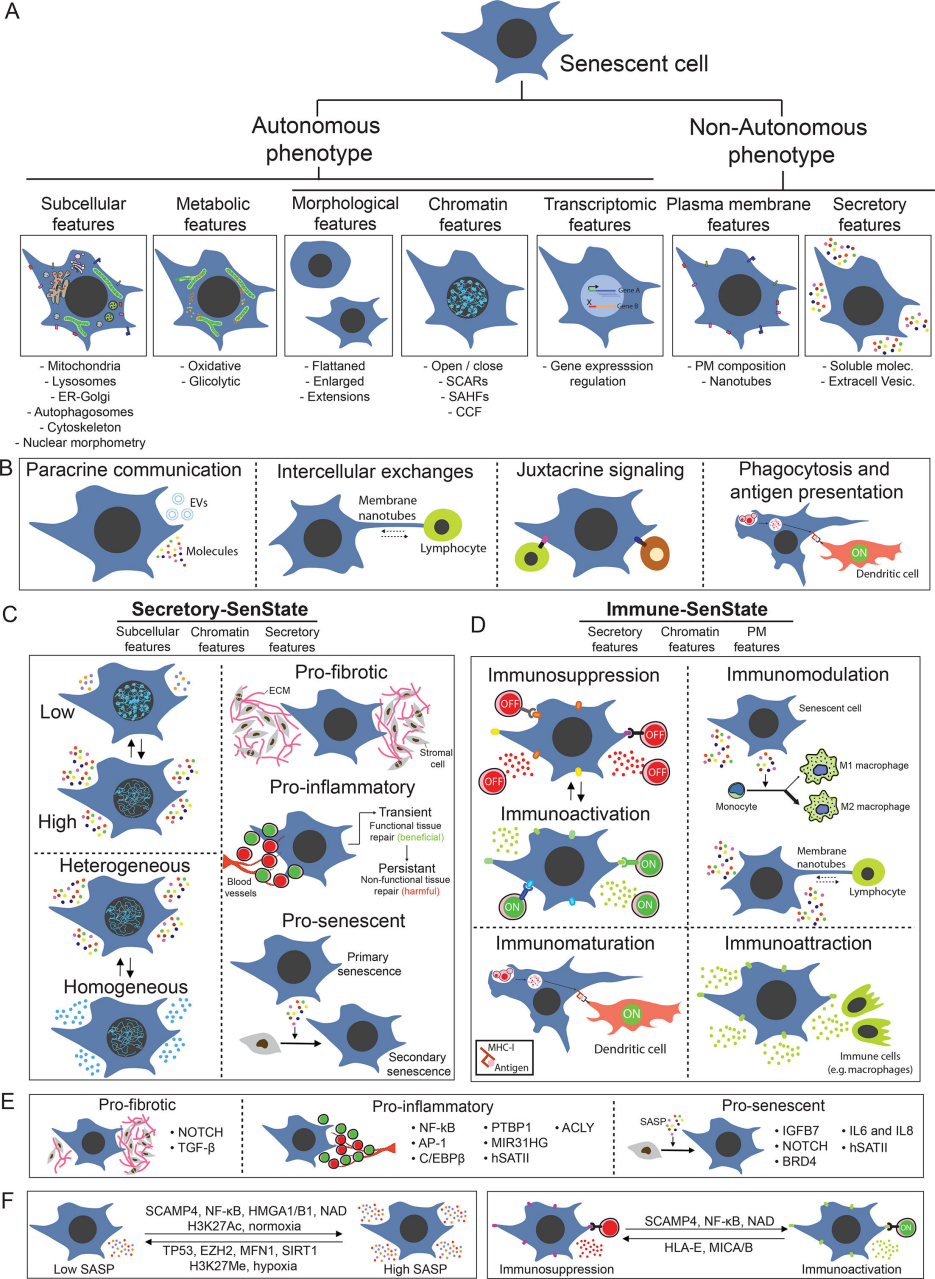
The states of senescent cells (SenStates). (**A**) General features and phenotypes of SnCs. (**B**) Mechanisms of intercellular communication established by SnCs. (**C and D**) Potential characteristics and biological effects of (**C**) Secretory-SenState and (**D**) Immune-SenState. (**E**) Main biological roles and molecular players controlling different Secretory-SenState. (**F**) Left – molecular players involved in the transition between low and high levels of SASP production; right – molecular players involved in Immune-SenState variations (i.e. immunosuppression or immunoactivation). Note: PM: Plasma Membrane. SASP: Senescence-Associated Secretory; Phenotype. ER: Endoplasmic Reticulum; SCARs: Senescence Associated Chromatin Rearrangements; SAHFs: Senescence-Associated Heterochromatin Foci; CCF: Cytoplasmic Chromatin Fragments; PM: Plasma Membrane; EVs: Extracellular Vesicles. ECM: Extracellular Matrix

Senescent cells (SnCs) have structural changes like increased cellular and nuclear size, cytoskeleton and plasma membrane alterations, and morphofunctional changes in organelles [2]. These features, added to the non-proliferative character, constitute the autonomous phenotype of SnCs. Despite not proliferating, however, SnCs are metabolically active and secrete many soluble molecules and extracellular vesicles, constituting the senescence-associated secretory phenotype (SASP), which characterizes the non-autonomous phenotype of SnCs (Figure 1A) [3]. Hundreds of soluble molecules are found in the SASP, including growth factors, interleukins, and factors promoting cellular transformation and plasticity [4]. Mainly through the SASP, SnCs contribute to physiological processes like development [5–8], tissue remodeling and regeneration [9–11], and wound healing [7,12,13]. SnCs also affect pathological contexts such as chronic inflammation (inflammaging) [14–16], metabolic diseases [17,18], cancer [19], osteoarthritis [20], and others [21]. In these scenarios, the effect of SnCs depends on variables like SASP constitution and the time SnCs remain in the tissue [22]. SnCs also exert biological effects through other cellular mechanisms like direct contact with neighboring cells, including membrane nanotubes [23–25] (Figure 1B). Establishing the non-autonomous communicative phenotype requires several changes from gene expression to cytoskeleton dynamics, modifications in plasma membrane, and cellular secretion. Although the understanding of SnCs has increased, some basic features still need to be better understood, such as intercellular heterogeneity, phenotypic plasticity, and cellular states. Considering their variability, SnCs are very heterogeneous for features like morphometry [26], intracellular compartments [27], transcriptome [28–30], pro-survival pathways [31], and senolytics sensitivity [32,33]. The SASP also varies between SnCs of different origins or induced by different stresses [4,22,34]. In contrast with intercellular heterogeneity, understanding plasticity and the functional states of SnCs is incipient. In this review, we explore the possible states of SnCs (SenStates) and discuss the molecular mechanisms involved in their maintenance and transition. We also argue about translational aspects and raise the main open questions in the field.

## SenStates: the states of SnCs

Given that literature often uses ‘cell phenotype’ and ‘cell state’ indiscriminately, we must first differentiate these concepts. Cell phenotype is an observable character, including physical appearance (e.g. size, shape, subcellular structure, and biochemical features) and behavior (e.g. proliferative, migratory, etc.), resulting from the interactions between genome, epigenome, and environment. Therefore, the phenotype of SnCs has two clinically relevant characteristics: a non-proliferative, growth factor-unresponsive feature (the autonomous phenotype), reflecting their intrinsic behavior; and their influence on neighboring cells and the extracellular matrix via secreted factors (the non-autonomous phenotype). The autonomous phenotype contributes to tumor suppression by halting proliferation, while the non-autonomous effects are more complex and depend on SASP composition. Each phenotype comprises numerous features, such as shape, size, subcellular structure, and chromatin organization (Figure 1A).

The cell state, in turn, is the integrated set of all phenotypes that a cell harbors at a given time. It can be considered a dynamic property associated with a specific transcriptome and proteome. Cell features can change during state transitions, including active promoters, expressed mRNAs, regulatory RNAs, translated proteins, cell shape, motility, and cell–cell interactions. Although defining what represents a state is challenging, a pragmatic approach is to characterize a different state whenever it has a relevant biological effect. Considering these concepts, we propose two SenStates: the ‘Secretory-SenState’ and the ‘Immune-SenState’. As mentioned, defining a cellular state required evidence demonstrating relevant effects exerted by the SnCs in pathophysiological conditions. It is also worth noting that specific features alone (e.g. cellular morphometry) can represent only phenotypic noise and not necessarily determine cellular states.

Finally, based on the stability of the cell cycle, senescence can also be defined as a cell fate. This definition is important because, unlike binary mechanisms (e.g. cell death or division), senescence is a progressive event that passes through a reversible (quiescence) to a permanent non-proliferative state. Noteworthy, here we will specifically discuss the states of SnCs without including previous states such as quiescence [35].

### Secretory-SenState

The main biological effects attributed to SnCs are played by SASP, which vary in intensity, composition, and effect (Figure 1C). Concerning the dominant biological impact, for instance, the SASP can be pro-fibrotic [36,37], pro-angiogenic [38,39], pro-inflammatory [40], or pro-senescent [41–43]. In some contexts, the role played by SASP is executed by dominant molecules, like Platelet-Derived Growth Factor-AA (PDGF-AA) in wound healing [12], Interleukin 6 (IL6) Interleukin 8 (IL8) and Insulin-like Growth Factor Binding Protein 7 (IGFBP7) in secondary senescence (i.e. senescence caused by SASP) [42,44], Vascular Endothelial Growth Factor (VEGF) for angiogenesis [38,39], IL6 in chronic inflammation [45,46], and Transforming Growth Factor Beta (TGF-β) for cell migration [47,48]. Therefore, because single or few molecules can be responsible for the main effect of SnCs, small variations in SASP can affect the biological role played by SnCs. However, more evidence is needed before generalizing the roles of specific SASP molecules across pathophysiological contexts, since many findings, though robust, come from specific models or cell types with unique features and SenState-specific differences.

SnCs can also vary SASP levels, so that SnCs can be in high or low Secretory-SenState. For example, senescent fibroblasts, tumor cells, and macrophages secrete high SASP levels, while mesenchymal and NK cells secrete intermediate levels. Finally, melanocytes and keratinocytes produce low amounts of SASP [22]. SASP levels also vary depending on the senescence-inducing stimuli. The SASP from cells induced to senescence by oncogene overactivation or irradiation has a similar number of proteins despite the proteome varying across cell types. In contrast, the SASP produced by cells induced to senescence by proteotoxic stress is less varied. Furthermore, the number of exosomal proteins released by cells induced to OIS is almost five times greater than senescence induced by irradiation [4]. Interestingly, cells induced to senescence by the CDK4/6 inhibitor abemaciclib produce a moderate SASP enriched in TP53-dependent molecules but lacking NF-κB-driven components [49]. Likewise, compared with senescence induced by replicative stress or oxidative damage, fibroblasts induced to senescence by proteasome inhibition also produce low levels of SASP, which is less variable and poorly inflammatory but strongly induces the proliferation of parental fibroblasts [50].

The variability of SASP composition likely reflects the diverse physiological roles of SnCs. With over 1500 soluble molecules and extracellular vesicles, SASP profiles vary by cell type, tissue origin, and senescence trigger, suggesting tissue context shapes SASP content toward beneficial functions. For instance, after injury, a SASP rich in ECM-modifying enzymes and growth factors may support tissue repair [4]. In severe damage, SASPs promoting plasticity, clearance, and matrix synthesis aid regeneration [11,51,52]. Additionally, chemokine-enriched SASPs may enhance immune responses [53,54]. This compositional flexibility underlies SnCs’ broad influence in development, repair, and immune modulation. Still, it’s possible SnCs lose transcriptional control, expressing atypical genes. Yet, selective increases in specific SASP factors imply some regulatory capacity [55], though the mechanisms remain unclear.

SASP levels may be modulated mainly by SASP-related transcription factors. Based on that, some authors have proposed the classification of SASP into (a) P-SASP (i.e. TP53-mediated SASP), which is triggered in response to DNA damage, endoplasmic reticulum (ER) stress, mitochondrial dysfunction, or CDK4/6 inhibition; and (b) N-SASP (i.e. NF-κB-SASP), which is mediated by Cyclic GMP-AMP synthase/Stimulator of Interferon Genes (cGAS/STING)/p38/NF-κB/IL1A pathway [49,56]. Even though N-SASP is typically inflammatory, and some senescence-inducing stimuli act mainly via P-SASP (e.g. CD4/6 inhibitors), there is a significant overlap of mechanisms (e.g. NF-κB) and secretomes between P-ASP and N-ASP, which also does not seem to be exclusive. Furthermore, both can be found in cells from the same cell population, and other players are also relevant to SASP establishment [22], like chromatin structure and organization. For instance, Enhancer of Zeste Homolog 2 (EZH2), a methyltransferase, promotes the heterochromatization and down-regulation of SASP genes [57]. Other elements of the protein secretion machinery may regulate SASP levels [58], including Ca^2+^ homeostasis via the ER and mitochondria [59,60], ER/Golgi stress pathways [61], microtubule integrity and dynamics [62], and microtubule-associated motor proteins [63].

Therefore, multiple players can control the main phenotypes of Secretory-SenState [22,64]. Modulating these players using molecules called senomorphics has great translational potential in physiopathological contexts [64]. Alternatively, attenuating the SASP effects can also be achieved using specific neutralizing antibodies that target individual SASP factors. Finally, the SASP constitution and levels are dynamic in cellular populations enriched in SnCs [22,65]. However, these analyses are population-based, and no evidence shows that the same SnC can change its Secretory-SenState over time.

### Immune-SenState

The second biologically relevant SenState concerns the ability of SnCs to modulate the immune system, here called Immune-SenState. This effect involves (a) secreted soluble molecules, (b) the release of exosomes, (c) direct contact between SnCs and immune cells, and (d) the formation of plasma membrane nanotubes between SnCs and immune cells. Through that, SnCs can activate or suppress the immune response (Figure 1D).

The most relevant driver of pro-inflammatory SASP is NF-κB [66], which encodes molecules like IL6, IL8, and IL1B, which are the main effectors of immune activation mediated by SnCs [67]. SnCs can also recruit immune cells by releasing chemokines, mainly from the CXC motif chemokine ligand family (CXCL), like CXCL8, CXCL10, and CXCL12 [68,69]. SASP molecules can also influence the immune system maturation [58,64,65] and macrophage polarization [68,70]. Beyond SASP, other mechanisms are relevant for determining the Immune-SenState. For instance, SnCs can phagocytize other cells [71–73], increasing Major Histocompatibility Complex-II (MHC-II) expression and the molecular machinery required for antigen processing and presentation [73–78]. Through that, SnCs can efficiently activate immune responses in the tumor microenvironment (TME) [75–77]. Finally, SnCs can activate specific immune cells like Natural Killers (NK) or cluster of differenciation 8 cells (CD8) by increasing surface markers like the Natural Killer Group 2D ligand (NKG2D), Major Histocompatibility Complex Class I-related chain A/B (MICA/B) [5,79], CD107, and Interferon Ɣ (IFN-Ɣ) [69].

5,79 69

On the other hand, SnCs can also negatively modulate immune responses through the direct contact with immune cells like NK and CD8 cells, the main effectors of eliminating SnCs in tissue [80]. Molecularly, SnCs can up-regulate plasma membrane immune inhibitory proteins, such as HLA-E [7], CD47 [81], and INHBA [82]. Additionally, SnCs can negatively modulate immune cells’ activity by establishing membrane nanotubes and directly transferring mitochondria [23] or releasing exosomes [83]. Finally, senescent gingival fibroblasts demonstrated lower interactivity and capacity to recruit peripheral mononuclear cells when compared with young fibroblasts. Furthermore, aged fibroblasts also had lower levels of MICA/B and CD112, two NK receptor ligands. Together, these features could affect the enrichment of immune cells in the microenvironment of injured tissues and, consequently, tissue repair in older individuals [84] beyond favoring the immunosurveillance of abnormal cells [85].

Considering that SnCs can suppress or activate immune responses, it is worth noting that inhibitory signals usually have a dominant effect on immune activity. Therefore, it is plausible to assume that the modulation of immune cells by SnCs influences the cytotoxic response in general and not just the immunosurveillance over the SnC itself, as discussed in SenState transitions, health and disease.

### Other potential SenStates

Based on the heterogeneity and the morphofunctional complexity of SnCs, other relevant SenStates may exist. For instance, SnCs present wide morphometric pleomorphism, from cells with an enlarged flat phenotype to protrusions-enriched elongated cells. Although cellular morphometry alone is not enough to define a SenState, morphological changes must be required to processes necessary for SenState establishment, like phagocytosis, antigen presentation, or the formation of plasma membrane nanotubes. Furthermore, in some scenarios, the cellular function is exercised by the cellular morphology itself, as in the network formed by reticular cell extensions in immune organs, which decelerate lymph or blood circulation, allowing greater access to the circulating contents by immune cells. A recent article proposed 11 subtypes of senescent fibroblasts based on 15 characteristics of individual SnCs, such as molecular, metabolic, and morphometric markers. However, despite variations in the prevalence of specific subtypes in response to senescence-inducing stresses, biological functions played by subtypes of SnCs were not investigated [26]. A significant limitation also concerns the difficulty of measuring cellular morphometry in tissue, especially including characteristics such as cell shape, cell volume, or multiple cell processes.

### SenStates: conclusions and perspectives

**Figure 2:**
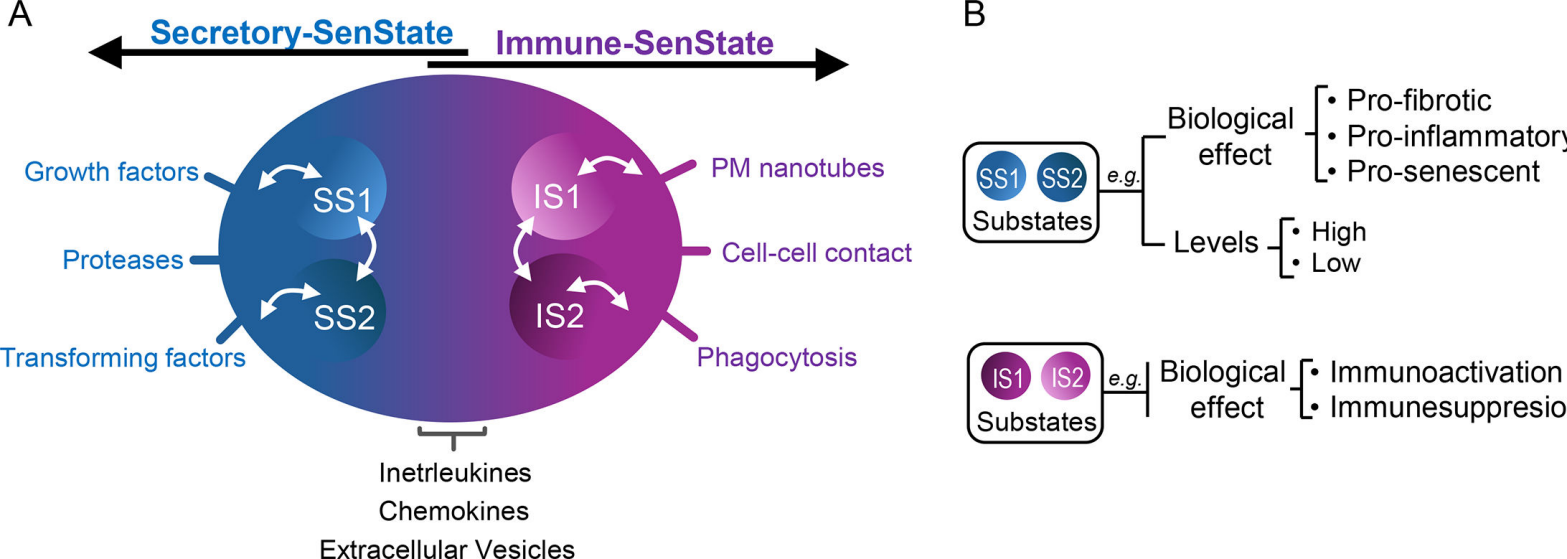
Intersection between Secretory and Immune-SenStates. **(A**) Each proposed SenState is mediated by specific molecules or mechanisms. However, some soluble molecules, such as interleukins and chemokines, in addition to extracellular vesicles, which are part of the Secretory-SenState also play biological roles related to the Immune-SenState. The arrows indicate that there may be a transition between the proposed substates (SS, secretory-substate; IS, immune-substrate). (**B**) Specific potential ‘substates’ within each of the SenStates.

In conclusion, it is plausible to infer that a SnC can inhabit different states with biological relevance. Certainly, even if one state is dominant over the other, there is a possible overlap or complementation between the SenStates caused, for example, by players shared by the different states, such as interleukins, chemokines, and extracellular vesicles (Figure 2A – bottom). On the other hand, some specific characteristics with a potential dominant role over the senescent cellular state could direct a more exclusive biological effect to the Secretory-SenState or the Immune-SenState (Figure 2A). For example, it is likely that a highly secretory SnC also has immunological impacts, and variations in the Immune-SenState may involve changes in the composition and levels of the Secretory-SenState (Figure 2A). If there is increasing evidence describing the transcriptomic heterogeneity of SnCs, these studies lack assessments of the biological function played by SnCs. To date, no evidence has integrated multiple characteristics of individual SnCs into comprehensive analyses of phenotypic dynamics and cellular states over time. SnCs may also exhibit substates – Secretory (SS1 and SS2) and Immune (IS1 and IS2) – with distinct morphofunctional traits tied to dominant roles (Figure 2A). For instance, SnCs might shift from pro-inflammatory to pro-fibrotic, or from immunoinhibitory to immunostimulatory substates. While transitions substates may be rapid and adaptive, shifts between major states may be more complex (Figure 2B). Molecular regulators or ‘switchers’ guiding these transitions will be discussed in Section 3, while Section 4 explores their translational relevance in human disease.

Although limited to populational information, initial evidence demonstrates the dynamics of autonomous and non-autonomous characteristics of SnCs [41,86]. Conversely, whether the varied transcriptome of SnCs in each cell population is due to different SnCs or whether the same SnC modifies its transcriptome over time remains unknown. It is also impossible to affirm which transcriptomic variations affect the state of SnCs. Despite these challenges, evidence has found key players relevant to the establishment and variations in SenStates, as discussed in the next section.

## Mechanisms controlling SenStates and SenStates transitions

Advances in understanding cellular states have been driven by improved techniques and equipment like single-cell analysis and live-cell imaging, image resolution and detection, and the development of cell reporters/probes and markers [87,88]. Mechanisms driving the establishment and dynamics of cell states involve mainly (a) the regulation of gene expression at a transcriptional level, including epigenetic and chromatin structure, and (b) the proteostatic phenotype, including protein translation and degradation and post-translational modifications. In this section, we explore the mechanisms and players controlling the key aspects of SenStates, including its transitions. We summarize the main players and their effects on SenState-related features in Table 1.

**Table 1:** Molecular players controlling SenState-associated features.

Molecular player	SenState	Senescence inducer	Cell model	Effect on senescence state	Ref
H3K9me2 and H3K27me3	Secretory	DDIS (TIS)	Tumor cell lines	Combined inhibition blocked cGAS-STING pathway promotes low SASP	[89]
PTBP1	Secretory and immune	OIS (NRAS)	Murine hepatocyte	Depletion of PTBP1 results in low SASP and poor immune recruitment	[90,91]
SCAMP4	Secretory and immune	RS	Fibroblast	SCAMP4 increases inflammatory SASP	[92,93]
NF-κB	Secretory and immune	RS	Primary cardiac fibroblast	NF-κB increases inflammatory SASP	[86,94]
NOTCH1	Secretory and immune	OIS (NRAS)	Murine hepatocyte	Low NOTCH1 activity increases inflammatory SASP and recruitment of immune cells; promotes TGF-β secretion and secondary senescence by cell-cell contact	[95]
AP-1/p300	Secretory	OIS	Fibroblast	Activate enhancers and super-enhancers that drive the expression of inflammatory SASP genes	[96,97]
HLA-E	Immune	DDIS	Primary human dermal fibroblasts	Immunosuppression by direct contact with NK and CD8	[7]
MICA/B	Immune	DDIS, RS, and OIS	Fibroblast	Immunosuppression by direct contact with NK and CD8	[5,76]
HMGA1	Secretory	OIS	Fibroblast	HMGA1 depletion up-regulates SASP inflammatory genes and down-regulates fibrogenic genes	[98]
					
H3K27me3/EZH2	Secretory and immune	DDIS	PDAC	EZH2 inhibition increases SASP inflammatory genes and immune recruitment.	[99].
BRD4/H3K27Ac/ ACLY	Secretory and immune	OIS (HRAS)	Fibroblast	BRD4 enhances inflammatory SASP and paracrine senescence induction. Inhibition is associated with reduced macrophage polarization and low NK activity.	[100]
HMGB2	Secretory	OIS (RAS)	Fibroblast	HMGB2 depletion results in inflammatory SASP.	[101]
lncRNA MIR31HG	Secretory	OIS (BRAF)	Fibroblast	Up-regulation is associated with inflammatory SASP.	[102]
lncRNA hSATII	Secretory	DDIS	Fibroblast	hSATII is associated with chromatin remodeling and increased expression of SASP genes.	[103]
AMPK activation/ mTOR inhibition	Secretory	DDIS	Primary human skin fibroblast and mice	Hypoxia induces AMPK activation and mTOR inhibition and suppress SASP inflammatory signaling	[104,105]
SIRT1	Secretory	RS, DDIS, and OIS	Primary human lung fibroblast	Autophagy degradation of SIRT1 promotes SASP inflammatory genes activation	[106]
TNIP1 / NF-κB	Secretory	DDIS	Fibroblast	Autophagy degradation of TNIP1 enhances NF-κB activity, leading to inflammatory SASP up-regulation	[92]
NAD^+^/NADH ratio	Secretory	OIS (RAS)	Fibroblast	High NAD^+^/NADH ratio enhances NF-κB activity, increasing inflammatory SASP	[107,108]

ACLY, ATP-citrate lyase. DDIS, DNA-damage induced senescence. TIS, Therapy Induced Senescence. OIS, Oncogene-Induced Senescence. RS, Replicative Senescence.

### Gene transcription

The main transcription factors controlling the expression of SASP-associated genes are NF-κB [87], CCAAT/enhancer binding protein beta (C/EBPβ) [88], neurogenic locus notch homolog protein 1 (NOTCH1) [109], GATA4 [110], Proto-oncogene MYB (c-Myb) [88], and TP53 [3]. Except for TP53, the other factors are associated with increased SASP levels. Furthermore, while some transcription factors do not appear to produce a specific SASP, others mediate the production of a SASP with a more inflammatory or fibrotic effect, like NOTCH1 and NF-κB.^109^ ^110^ ^88^ ^3^

NF-κB is a central regulator of the inflammatory SASP [86]. Activating the cGAS/STING/NF-κB pathway allows cells to detect cytoplasmic DNA, such as cytoplasmic chromatin fragments (CCFs) [111]. The accumulation and detection of CCF in SnCs play a significant role in inducing inflammatory SASP genes [111–114]. Translationally, blocking this pathway suppresses SASP production by senescent hepatic stellate cells and reduces the development of obesity-associated hepatocellular carcinoma *in vivo* [111]. Additionally, mitochondrial double-stranded RNA (mt-dsRNA) released into the cytosol drives pro-inflammatory responses by activating pathways such as RIG-I/MDA5/MAVS/NF-κB. Targeting EZH2 [57], which regulates CCF formation, and MFN1, involved in mt-dsRNA release, can effectively reduce SASP production, offering potential targets to mitigate senescence-mediated inflammation [55].

NOTCH is another family of SASP regulators [95,109]. High NOTCH activity is associated with a pro-fibrotic secretome enriched in TGF-β and other growth factors, supporting tissue repair and regeneration. In an Neuroblastoma Rat Sarcoma (NRAS)-induced murine hepatocyte senescence model, NOTCH1 signaling represses C/EBPβ, a key transcription factor regulating pro-inflammatory SASP [115,116]. This repression is essential for maintaining the immunosuppressive and pro-fibrotic functions of the secretome during the early stages of senescence. Conversely, low NOTCH1 activity leads to the activation of the C/EBPβ pathway, resulting in the transcription of pro-inflammatory SASP components, enhancing the recruitment of immune cells, and the immune-mediated clearance of SnCs [115]. This is a representative example of a molecular switch that could control the transition between senescent substates, as proposed in Figure 2B. NOTCH signaling also plays a pivotal role in triggering SASP-independent secondary senescence through cell–cell interactions, called NOTCH-induced senescence (NIS). Unlike OIS, which is characterized by low NOTCH activity, increased activity of the C/EBPβ and a pro-inflammatory SASP, NIS exhibits a low secretory profile, lacking inflammatory components and, instead, being enriched with fibrillar collagens. Furthermore, NIS is marked by TGF-β pathway activation, which promotes fibrotic and tissue-remodeling properties [117]. Thus, NOTCH acts as a molecular switch, balancing tissue repair and immune surveillance by modulating the role played by the Secretory-SenState.

Finally, Activator Protein 1 (AP-1) is another transcription factor regulating the Secretory-SenState. It binds to enhancers and promoters, working with histone acetyltransferases like p300 to modify chromatin structure, enhancing the expression of inflammatory SASP genes [118]. In prostate cancer, AP-1-mediated SASP is essential for recruiting immune cells to the TME, promoting the clearance of SnCs and suppressing tumor progression. Additionally, inhibiting AP-1 can reverse the pro-inflammatory phenotype associated with senescence without affecting the cell cycle arrest, further emphasizing AP-1’s pivotal role in controlling the Secretory-SenState without affecting the autonomous phenotype of SnCs [118], besides potentially contributing to transitions between senescent substates (Figure 2B). In *Drosophila melanogaster*, c-Jun N-terminal kinase (JNK)/AP-1 signaling induces the enrichment of SnCs in wound-center cells, which then secrete mitogenic ligands to mediate tissue regeneration. In this scenario, JNK/AP-1-positive cells secrete Unpaired (Upd) cytokines, which are responsible for binding to the Dome receptor and activating the Janus kinase/Signal Transducer and Activator of Transcription (JAK/STAT) pathway in *Drosophila melanogaster*. Consequently, JAK/STAT signaling is activated in neighboring cells, while AP-1 simultaneously suppresses JAK/STAT activity within the cells that secreted Upd. This ensures that these cells act solely as signal senders, promoting the proliferation of nearby cells without responding to their own mitogenic cues. Such mutually exclusive regulation maintains a strict division between senescent and proliferative states, supporting organized tissue regeneration and highlighting AP-1’s central role in establishing the Secretory-SenState [119].

In conclusion, some transcription factors are crucial in determining the SenStates, even though how SnCs control these factors remains unknown. Several transcription factors and associated pathways must be active in the same SnC so that the SASP composition and its dominant effect result from various transcriptional events. Therefore, these players are potential targets for senomorphics (i.e. agents that modulate SASP composition), which can be considered a strategy to modulate the SenStates.

### Epigenetic mechanism

The establishment and maintenance of cellular senescence are finely regulated by epigenetics, including chromatin modifications and remodeling [120]. Among these modifications, histone acetylation and methylation regulate gene accessibility by acting as signals for transcription factor recruitment or chromatin compaction [55]. For example, H3K9me3, a constitutive histone repression mark, is associated with senescence-associated heterochromatin foci (SAHF) formation in OIS. Constitutive H3K9me3 and facultative H3K27me3 are known to induce cell cycle inhibitors like p21 and p16 [90]. Epigenetic marks particularly influence the Secretory-SenState [55]. For instance, H3K27 acetylation (H3K27Ac) is associated with the activation of SASP-related enhancers [117], while H3K27me3 leads to transcriptional repression of SASP genes [99]. Adding complexity to this control, the dual inhibition of H3K9me2 and H3K27me3 can promote tumor cell senescence without triggering SASP by blocking CCF formation and cGAS/STING activation [89]. This suggests that epigenetic regulation can be leveraged to induce senescence without the harmful consequences of SASP.

A major epigenetic player regulating the Secretory-SenState is the Polycomb Repressive Complex 2, which mediates gene silencing through H3K27me3. Its catalytic component, EZH2, suppresses the expression of cell cycle and SASP genes. In pancreatic ductal adenocarcinoma cells, EZH2 fosters an immunosuppressive TME by repressing SASP genes. At the same time, its inhibition reprograms SASP, shifting it toward a pro-inflammatory phenotype that enhances immune surveillance and promotes tumor regression [121]. Moreover, BRD4, a chromatin reader, facilitates SASP gene expression and mediates paracrine senescence. Inhibition of BRD4 disrupts SASP-driven paracrine effects, compromises immune surveillance, and reduces macrophage polarization and NK cells’ activity, underscoring its role in determining SenStates [122].

Epigenetic control of SenStates also extends to alternative RNA splicing. Polypyrimidine Tract Binding Protein 1 (PTBP1), a splicing factor influencing exon skipping, is a critical regulator of pro-inflammatory SASP [91]. PTBP1 depletion leads to the inclusion of exon 7 in EXOC7, generating a long isoform (EXOC7-L) that is less effective in promoting SASP than the short isoform (EXOC7-S). This SASP reduction compromised the immune infiltration, illustrating how alternative splicing can finely regulate SenStates. Non-coding RNAs (ncRNAs) also regulate senescence at transcriptional, post-transcriptional, and post-translational levels. In B-homolog Rapidly Accelerated Fibrosarcoma (BRAF)-induced senescence, lncRNA MIR31HG is up-regulated and interacts with YBX1 in the cytoplasm, enhancing its phosphorylation (p-YBX1S102) via Ribosomal S6 Kinase (RSK). This modification promotes IL1A translation, activating other pro-inflammatory SASP genes such as IL6 and CXCL1. Notably, MIR31HG depletion reduces SASP cytokine expression without affecting paracrine senescence or TGF-β signaling, indicating that its role is specific to regulate inflammatory SASP genes rather than broadly controlling SASP [102]. Finally, hSATII, an ncRNA, alters the DNA-binding activity of CCCTC-binding factor, a major chromatin architecture regulator, enhancing the expression of inflammatory SASP genes. Additionally, hSATII is detected in extracellular vesicles secreted by SnCs, suggesting a role in paracrine senescence by transferring senescence cues to neighboring cells [103].

In summary, epigenetic regulation of SenStates involves a complex interplay of histone modifications, chromatin remodeling, transcription factor recruitment, alternative splicing, and ncRNA-mediated control. Collectively, these mechanisms shape SenStates and SASP dynamics, including potential transitions between senescent substates (Figure 2B), offering potential therapeutic strategies to modify the biological effects played by SnCs.

### Chromatin structure

Alterations in key aspects of nuclear reorganization, such as nuclear size, DNA–lamin interactions, SAHF formation, and chromatin composition, have been linked to cellular senescence [94,123]. Particularly, changes in chromatin structure are closely associated with distinct SenStates, a dynamic that may contribute to changes in senescent substates (Figure 2B). Active chromatin was linked to up-regulated SASP genes, while repressed chromatin correlated with silenced genes like those involved in cell cycle progression. Surprisingly, the primary chromatin modifications described in senescence occurred mainly at enhancers, which transitioned between poised and active states, whereas promoter regions remained stable. Notwithstanding, the kinetics of chromatin transitions varied depending on the senescence inducer. In OIS, enhancer activation occurred within days, whereas, in RS, chromatin modifications progressed more gradually [97].

Chromatin-associated proteins are the most relevant players influencing chromatin reorganization in SnCs. HMGA1 is a chromatin-associated protein crucial in organizing higher-order chromatin architecture and regulating gene expression during senescence. In OIS, HMGA1-rich regions form highly interactive chromatin networks, particularly in association with H3K9me3-marked heterochromatin and SAHF. In regions with lower chromatin density, HMGA1 contributes to gene activation by allowing transcription factor accessibility. Its depletion leads to enhanced SASP expression and down-regulation of ECM components, suggesting that HMGA1 plays a dual role in both Secretory- and Immune-SenStates by regulating inflammatory and fibrogenic SASP genes [98]. HMGB1, which is reduced in SnCs compared with proliferative cells, also contributes to changes in 3D chromatin architecture and transcriptome, increasing the expression of SASP genes [124]. Finally, HMGB2 contributes to SASP gene expression by preventing heterochromatin expansion, while its loss results in SASP gene silencing due to its incorporation into SAHF [101].

Although there are gaps in understanding the structure and dynamics of chromatin in senescence and SenState establishment, initial evidence has suggested mechanisms and players showing the effects of chromatin modifications on SenStates. This evidence allows targeting specific regulators like HMG proteins and AP-1 to regulate SenStates and SenStates transitions to attenuate the deleterious effects of SASP or enhance its beneficial properties.

### Other mechanisms affecting SenStates

#### Tissue environment

One of the key environmental factors influencing SenStates is substrate stiffness. In senescent fibroblasts, high stiffness drives myofibroblast activation, leading to a pro-fibrotic SASP, while low stiffness up-regulates NF-κB-responsive genes, promoting a pro-inflammatory effect. Interestingly, once the pro-inflammatory SASP is established, it persists despite changes in substrate stiffness due to epigenetic ‘locking’ — the stabilization and maintenance of epigenetic features even after environmental conditions have shifted [125]. Oxygen availability also affects the Secretory-SenState. Normoxia promotes a high-SASP, pro-inflammatory state, while hypoxia suppresses SASP through AMP-Activated protein kinase (AMPK) activation and mTOR inhibition, reducing inflammatory signaling [104,105].

#### Autophagy

Autophagy is a physiological mechanism regulating protein turnover and stress responses, allowing damaged cells to avoid apoptosis and enter senescence [126]. Autophagy also actively contributes to the senescent phenotype by promoting LaminB1 degradation [127]. Selective autophagy contributes to establishing Secretory-SenState through the targeted degradation of regulatory proteins [128] like SIRT1, a repressor of SASP-related genes, thereby promoting SASP production [106]. Selective autophagy also degrades TNIP1, a negative regulator of the NF-κB pathway, leading to enhanced NF-κB activity and the activation of pro-inflammatory SASP [92]. Finally, the selective autophagy of GATA4, a transcription factor normally degraded by p62-mediated selective autophagy, acts as a switch to pro-inflammatory SenState by up-regulating NF-κB signaling [110].

#### Metabolic pathways

During cellular senescence, mitochondria undergo metabolic reprogramming, leading to the accumulation of metabolites such as acetyl-CoA, succinate, and fumarate [129]. Mitochondrial metabolism and associated pathways can modulate SASP gene expression and Secretory-SenState transitions [130]. For instance, ATP-citrate lyase converts citrate to acetyl-CoA, a histone acetylation substrate controlling the accessibility of transcription factors to SASP genes, mostly encoding pro-inflammatory molecules [40]. Mitochondria are also linked to mitochondrial dysfunction-associated senescence, which is characterized by moderated SASP lacking pro-inflammatory molecules. This phenotype is associated with a low NAD^+^/NADH ratio and AMPK-mediated TP53 activation, contributing to a distinct SenState [27]. Likewise, a decrease in the NAD^+^/NADH ratio activates AMPK, resulting in TP53 activation and p38 and NF-κB suppression, reducing SASP levels. Complementary, high NAD^+^ levels, controlled by the HMGA1-NAMPT axis, enhance NF-κB activity, promoting a pro-inflammatory SASP [107].

#### Plasma membrane proteins

Beyond intercellular communication, plasma membrane proteins also regulate SASP secretion. SCAMP4, a cell surface protein, is rapidly degraded via the ubiquitin-proteasome pathway in proliferating cells. In SnCs, however, it accumulates at the plasma membrane, leading to increased secretion of SASP by unknown mechanisms. Silencing SCAMP4 reduces the production of SASP cytokines such as IL8, MIF, and IL1B, while its overexpression is sufficient to increase SASP secretion, mainly IL1B and IL8 [93].

## SenState transitions, health, and disease

Although multiple evidence has shown the influence of SnCs in physiopathological processes, data demonstrating a biological role for transitions between SenStates, as proposed in Figure 2, are still scarce or even absent. This gap is due to technical and biological challenges and limitations, such as (a) the difficulty of following the same SnC long enough to detect changes in cell state, including co-culture systems with other cells; (b) the difficulty in detecting the production and secretion of specific SASP molecules by live SnCs; (c) the scarcity of SenStates markers; and (d) the uncertainty about the relevant SenStates. Despite these limitations, it is plausible to hypothesize that transitions between the SenStates, as raised in Figure 2B and for which potential players have been described (Figure 1E and F; Table 1), are relevant in human health and disease. It is worth noting that most studies on molecular mechanisms regulating senescent cell states use normal cells as models (Table 1). This suggests some regulatory features may be conserved across cell types, while others are origin-specific. Senescent tumor cells may exhibit a broader, more complex range of states and greater plasticity, reflecting neoplastic biology. Although tumor models have provided key insights, extrapolating their findings to senescent states requires caution. SnCs are recognized and eliminated by immune cells, so variations in Immune-SenState should modulate their lifespan and, consequently, their biological impact [131]. On the one hand, SnCs can express molecules suppressing the activity of CD8 or NK, the most important cells involved with SnCs clearance [131,132]. On the other hand, SnCs can express immunogenic molecules that recruit and activate NK cells [7,69]. Then, through the switch from immunosuppressive to immune-activating state, SnCs can exert their biological effect for a specific time, followed by their removal by immune cells [7,133]. This programmed SenState transition may be relevant because SnCs for longer than necessary cause harmful effects [134], like dysfunctional healing and fibrosis [135–137], beyond alterations in tissue homeostasis and regeneration [138–140]. At least partially, an imbalance in controlling the duration of SnCs in tissue occurs due to immunosenescence, the functional deterioration of the immune system that occurs with aging that favors SnCs’ accumulation in aged tissue and the subsequent loss of tissue function, creating positive feedback [138,141]. Environmental changes like obesity and inflammation can accelerate this process [142,143], thus potentially affecting tissue homeostasis.

Varying between immunosuppressive and immune-stimulating states can also affect the role of SnCs in diseases such as cancer [80]. While SnCs can be immunogenic and promote anti-tumor immunity in some conditions [144], in other contexts, immunosuppressive strategies favoring SnCs’ immune escape may also benefit tumor cells [143,145]. Consequently, SnCs may reduce the responsiveness to immunotherapy [146–148]. Additionally, changing the SASP can also switch the TME from inert to immune attractive [68,69,149], like senescent fibroblasts and macrophages, which can promote myeloid infiltration and inhibit T cell-mediated anti-cancer immunity [143]. Finally, SnCs can reshape the TME through other mechanisms [145–150] like macrophage polarization [68,70], CD8 lymphocyte activity [75], NK activity [7,132], and fibroblast functions [151–153]. Interestingly, even benign (hepatoma) SnCs can affect the plasticity of immune cells [70], suggesting an impact on SenStates throughout carcinogenesis.

Like Immune-SenStates, transitions between Secretory-SenStates, including changes in the quantity, content, and heterogeneity of the SASP, may also mediate physiological responses [22,134]. In healing, for example, there is a transient enrichment of SnCs for three to four days, followed by reduction to avoid dysfunctional fibrosis [12]. Factors controlling such dynamics are just being described, including transcription factors, epigenetic players, and plasma membrane proteins. For instance, a shift from early pro-fibrotic SenState to late pro-inflammatory SenState is regulated by NOTCH1 signaling [115]. In this process, early SnCs have elevated NOTCH1 activity, low levels of NAD, and low SASP enriched with TGF-β and growth factors [107,154]. Subsequently, SnCs change to an increased secretory state mediated by NF-κB, with low NOTCH1 activity, high levels of NAD, and increased inflammatory molecules [107]. This process appears to respond to different senescence-inducing stresses, including mitochondrial damage [108]. This transition could also mediate the early effect by SnCs on tissue repair and remodeling, followed by a long-term pro-inflammatory effect [116]. Finally, this dynamic appears to influence the distinct Secretory-SenState between primary and secondary senescence. Unlike primary senescence, secondary senescence is characterized by an attenuated SASP, with fewer inflammatory molecules and high levels of fibrillar collagens [117], which may explain the deleterious effects of long-lasting senescence in tissues. Although several players involved in secondary senescence have been identified [155], little is known about the features of secondary SnCs.

All evidence characterizes SenStates as dynamic conditions, with transitions influenced by intrinsic stress responses and external cues. Therefore, it is plausible to assume that two different mechanisms can mediate SenStates transitions. The first is the programmed SenState transition, mainly involved in physiological processes [156,157]. In these scenarios, the same SnC may modify its state over time, playing different roles before eventually being cleared by the immune system [7]. The second mechanism could be environment-induced SenState transitions. This process is facilitated or even possible due to alterations in plasma membrane features [96] and may occur mainly in disease-associated microenvironments in which SnCs accumulate. Inducers mediating environment-induced SenState transitions include soluble factors like TGF-β [158], neighbor cells [93,117], extracellular matrix [125], and oxygen availability [105]. In these contexts, paracrine disruption and increased phenotypic plasticity should modify several features of SnCs, eventually leading to SenStates that do not exist in physiological contexts.

It is possible that transitions between SenStates require changes to various cellular features. Whether through endogenous or exogenous stimuli, the integration of these changes would trigger SenState alterations. A better understanding of SenStates and how these states are established and modified, including differences in normal and tumor SnCs, could allow targeting SnCs in age and age-related diseases, including the development of senotherapies.

**Perspectives**

- Senescent cells (SnCs) influence development, tissue repair, and aging-related diseases. While their morphofunctional traits are well described, there’s a need to integrate these features into defined cellular states, which drive their biological effects. Understanding these states may uncover modifiable targets for senotherapies, i.e. interventions that regulate senescent cell function.
- SnCs are heterogeneous and phenotypically dynamic, occupying various states, especially secretory and immune-modulatory, which evolve and produce distinct outcomes. These states are regulated by factors like transcriptional programs, epigenetic changes, and chromatin status.
- Future efforts should refine the concept of SenStates. Determining whether population heterogeneity reflects state changes within single cells over time is essential, while current data on state dynamics within individual SnCs are limited. Moreover, while SenStates transitions are plastic and environmentally driven, the reversibility and modulation of these states remain unclear. Unraveling these mechanisms could yield new therapeutic targets for managing aging and disease, reinforcing beneficial effects or attenuating harmful impact played by SnCs.

## Open Access

This article has been published open access under our Subscribe to Open programme, made possible through the support of our subscribing institutions, learn more here: https://portlandpress.com/pages/open_access_options_and_prices#conditional

## Abbreviations

AP-1: Activator Protein-1
CCF: cytoplasmic chromatin fragment
DDIS: DNA-damage Induced Senescence
ER: endoplasmic reticulum
H3K27Ac: H3K27 acetylation
NIS: NOTCH-induced senescence
OIS: Oncogene Induced Senescence
OIS: oncogene-induced senescence
SAHF: senescence-associated heterochromatin foci
SASP: senescence-associated secretory phenotype
SenStates: Senescence States
SnCs: senescent cells
SnCs: Senescent Cells
TIS: Therapy Induced Senescence
TME: tumor microenvironment
mt-dsRNA: mitochondrial double-stranded RNA
ncRNAs: non-coding RNAs
